# Supplementary material for: Saccharomyces boulardii (CNCM I-745) improves intestinal damage in sepsis by remodeling intestinal flora structure
Source: Front Cell Infect Microbiol. 2025 Jul 31;15:1602792. doi: 10.3389/fcimb.2025.1602792 (PMC12350329; doi:10.3389/fcimb.2025.1602792)
Supplement: Supplementary file 1 [file DataSheet1.zip › summary/summary/7_Differential_analysis/2_LEfSe_result/Group_CW0_vs_AW0_0.05_3.0/LEfSe_Group_CW0_vs_AW0.cladogram.pdf]

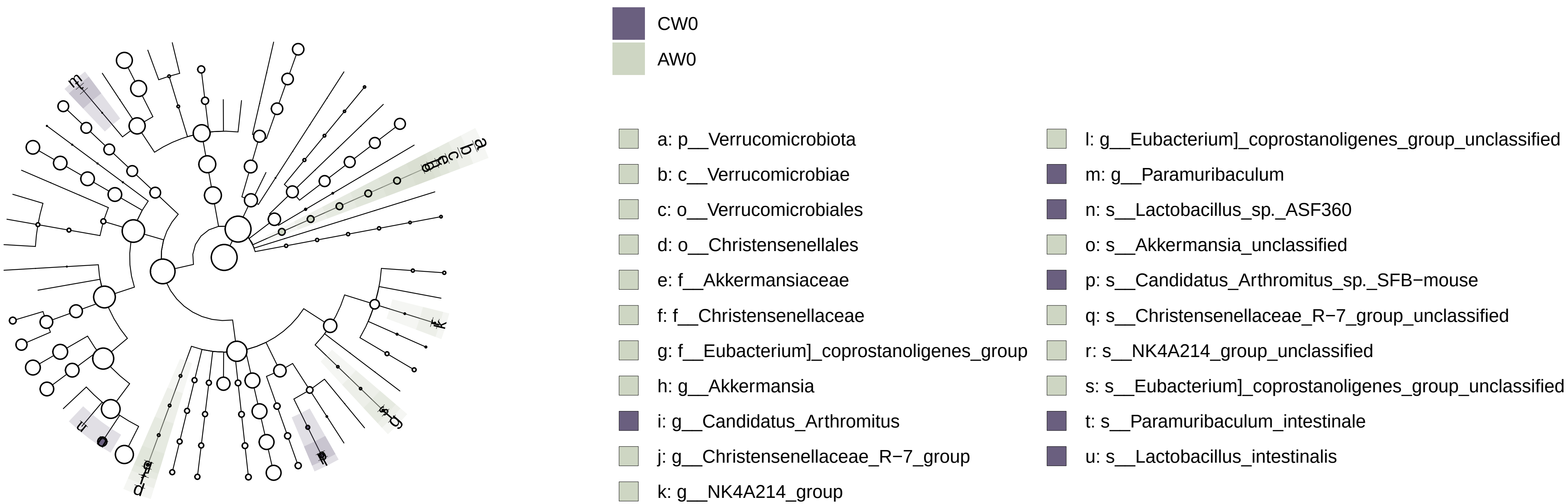
CW0
AW0
a: p__Verrucomicrobiota
b: c__Verrucomicrobiae
c: o__Verrucomicrobiales
d: o__Christensenellales
e: f__Akkermansiaceae
f: f__Christensenellaceae
g: f__Eubacterium]_coprostanoligenes_group
h: g__Akkermansia
i: g__Candidatus_Arthromitus
j: g__Christensenellaceae_R−7_group
k: g__NK4A214_group
l: g__Eubacterium]_coprostanoligenes_group_unclassified
m: g__Paramuribaculum
n: s__Lactobacillus_sp._ASF360
o: s__Akkermansia_unclassified
p: s__Candidatus_Arthromitus_sp._SFB−mouse
q: s__Christensenellaceae_R−7_group_unclassified
r: s__NK4A214_group_unclassified
s: s__Eubacterium]_coprostanoligenes_group_unclassified
t: s__Paramuribaculum_intestinale
u: s__Lactobacillus_intestinalis
